# Improvement of Quality of Sour Camel Milk by Extract of *Sparassis crispa*: Physicochemical Properties, Sensory Quality and Metabolic Changes

**DOI:** 10.3390/foods14173042

**Published:** 2025-08-29

**Authors:** Lina Zhao, Ruping Ma, Linyan Zhu, Jinzhi Wang, Rui Wang, Xiaojun Wu, Xiaoyan Liu, Xinhong Huang, Lianchao Zhang, Bin Liu

**Affiliations:** 1 National Engineering Research Center of JUNCAO Technology, Fujian Agriculture and Forestry University, Fuzhou 350002, China; 18806086183@163.com (R.M.); zhuly2024@163.com (L.Z.); 15939735893@163.com (J.W.); 18837610998@163.com (R.W.); xjun1013@163.com (X.W.); 2 College of Life Sciences, Fujian Agriculture and Forestry University, Fuzhou 350002, China; 3 Beijing Engineering and Technology Research Center of Food Additives, School of Food and Health, Beijing Technology and Business University, Beijing 100048, China; liuxiaoyan8112@163.com; 4 Xinjiang Camel Milk Engineering Technology Research Center, Yining 835100, China; huangxinhong0916@163.com (X.H.); lzh130227@163.com (L.Z.); 5 College of Food Science, Fujian Agriculture and Forestry University, Fuzhou 350002, China

**Keywords:** *Sparassis crispa*, sour camel milk, texture, metabolomics

## Abstract

Sour camel milk, as a nutritious fermented dairy product, faces challenges in terms of quality stability. *Sparassis crispa*, due to its antioxidant and antibacterial properties, shows potential in improving food quality. This study aimed to investigate the effects of different active components of *Sparassis crispa* on the quality of sour camel milk. The results indicated that Component I was the most effective *Sparassis crispa* component in enhancing the quality of sour camel milk. The components of Component I were identified as LysoPC(0_0_18_2(9Z,12Z)), LysoPC(18_1(11Z)_0_0), and N-(2-hydroxymethyl-3-chloro-4-hydroxyphenyl) anthranilic acid, among others. It increased the total viable count of lactic acid bacteria (LAB) and water-holding capacity (WHC) while improving the texture of sour camel milk. Metabolomics analysis revealed that the first component of sour camel milk (FCS) and *Sparassis crispa* sour camel milk (SS) have a high degree of similarity in the composition of flavor substances. The characteristic flavor metabolites included 2-amylfuran, isoamyl alcohol, 2-methylbutyraldehyde, and 2-ethyl-1-hexanol. Additionally, the supplementation of Component I increased the levels of metabolites such as amino acids, free fatty acids, organic acids, and carbohydrates, thereby contributing to the enhanced taste and nutritional quality of sour camel milk. This intervention also strengthened carbohydrate and amino acid metabolism in LAB. These findings provide a theoretical basis for utilizing Component I to improve the quality of sour camel milk.

## 1. Introduction

Sour camel milk is a product made from raw camel milk or reconstituted camel milk under aseptic conditions. It is subjected to fermentation using specific starter cultures, which leads to a decrease in its pH value. Sour camel milk is rich in nutrients such as lactose, protein, fat, vitamins, and trace elements [1]. It exhibits high nutritional value and health benefits [2]. Extensive studies have demonstrated its benefits, including improving intestinal flora, lowering cholesterol and blood sugar levels, enhancing immune function, and exhibiting auxiliary lifespan extension effects [3,4,5,6,7]. The viscosity of camel milk yogurt is only 60–70% of that of regular milk (compared before and after the addition of additives), the whey separation rate is as high as 25%, and the sensory score for odor decreases by 20% [8]. Due to the fact that some fatty acids and phenolic compounds in camel milk are prone to form odor or cause unstable quality, its usage is somewhat limited [9]. These problems need urgent improvement.

Research shows that adding natural plant resources rich in functional components can significantly enhance the health benefits of fermented milk [10]. Studies indicate that edible fungi serve as excellent prebiotics for probiotic growth. For instance, extracts from edible fungi, such as *Pleurotus ostreatus*, *Pleurotus eryngii*, and *Ganoderma lucidum*, have been shown to promote the growth of beneficial bacteria (e.g., *Bifidobacterium*) while inhibiting harmful pathogens (e.g., *E. coli*) [11,12,13]. Plant-based polysaccharides, proteins, and dietary fibers also promote the growth of probiotics and improve the physicochemical properties and stability of fermented milk [14,15]. *Sparassis crispa* polysaccharides can promote probiotic proliferation and improve the stability of yogurt, while suppressing pathogenic bacterial growth [16,17]. *Sparassis crispa* can also promote digestion, enhance immunity, and reduce fat [16,18,19]. At present, research on the effects of *Sparassis crispa* in fermented milk predominantly focuses on its polysaccharides, while the impact of other *Sparassis crispa* components remains understudied, especially in sour camel milk.

Previously, our laboratory discovered that *Lactobacillus acidophilus* (ATCC 4797), Lactobacillus delbrueckii subsp. bulgaricus and Streptococcus thermophilus (CICC 20370) exhibit a synergistic effect in promoting the fermentation of sour camel milk. In this study, *Sparassis crispa* was incorporated into acidic camel milk, aiming to enhance the flavor and stability of camel milk-based dairy products. The straightness of the acid camel milk was evaluated by measuring key indicators such as the survival rate of lactic acid bacteria, acidity, texture, rheology, and sensory properties. Metabolomics further analyzed the volatile and non-volatile metabolites in FCS, and also examined the effects produced by the metabolic pathways. This study aims to provide a theoretical foundation and data-driven support for the development of sour camel milk incorporating edible fungi.

## 2. Materials and Methods

### 2.1. Strain Activation

*L. acidophilus* (No. ATCC 4797), *L. delbrueckii* subsp. *bulgaricus*, and *S. thermophilus* (No. CICC 20370) were obtained from Beijing Microbiological Culture Collection Center. Each strain was cultured in MRS broth (BS1138, Hangzhou Baisi) for 24 h and subcultured to the third generation.

### 2.2. Preparation of Sour Camel Milk

**Figure 1:**
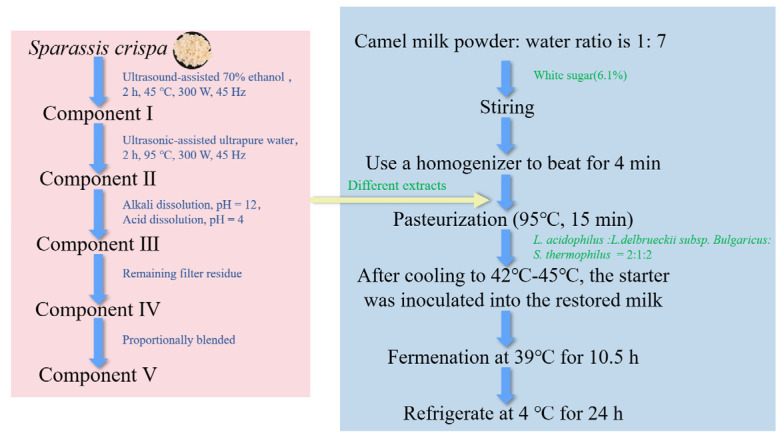
The general process for producing sour camel milk by adding *Sparassis crispa* extracts obtained through different extraction techniques.

*Sparassis crispa* was purchased from the Qingshitang flagship store. The preparation of *Sparassis crispa* is shown in Figure 1. The ratio of the material to the liquid was 1:25, and the solvent was 70% anhydrous ethanol. Ultrasonic-assisted extraction was carried out, and the filtrate was collected and freeze-dried to obtain Component I; the filtrate was collected from the above residue, the ratio of the material to the liquid was 1:25, and the solvent was water. The same treatment was carried out as in the previous part to obtain Component II; the filtrate was collected from the above residue, the ratio of the material to the liquid was 1:20, the pH was adjusted to 12, and it was left to stand overnight. The filtrate was collected, the pH was adjusted to 4, the precipitate was taken, and Component III was obtained; the filtrate of Component III was collected, and Component IV was obtained. Then, these fractions were mixed according to the extraction ratio (Component I, Component II, Component III, and Component IV were added in proportions of 29.39%, 7.54%, 2.49%, and 60.58%, respectively) to obtain Component V. All samples were sealed and stored at −20 °C.

Camel milk powder (Xinjiang Junong Ruye, Yining City, China), water, and sugar (Yunnan Zhezhentian Trading Co., Ltd., Kunming, China) were mixed thoroughly until completely dissolved, then different components of *Sparassis crispa* were added according to the extraction rate. The mixture was homogenized using a BILON-08 sterile homogenizer (Shanghai Bilang Instrument Co., Ltd., Shanghai, China). It was then heated in a water bath at 95 °C for 15 min. The mixture was then cooled down to 40~45 °C. A total of 7.1% of the designated starter culture (*L. acidophilus*: *L. delbrueckii* subsp. *bulgaricus*: *S. thermophilus* = 2:1:2) was accessed and incubated at 39 °C for 10.5 h under sterile conditions. It was refrigerated at 4 °C for 24 h to complete maturation (Figure 1).

### 2.3. Determination of Cell Counts

Total Plate Count (TPC) of lactic acid bacteria in the fermented camel milk was determined in accordance with the Chinese Standard (GB 4789.35-2016) [20]. The samples were serially diluted and inoculated onto MRS medium. The cultures were incubated anaerobically at 36 °C for 72 h, and the white colonies were counted. The results were expressed as LgCFU/mL, with the data presented as the mean value of three replicates ± standard deviation.

### 2.4. Determination of TA, pH, and WHC

The sour camel milk samples were reconstituted in distilled water at a 20% (*w*/*v*) concentration. The titratable acidity was determined by acid–base titration in accordance with the Chinese Standard (GB 5009.239-2016) [21]. The pH value was measured using an FE28 pH meter.

Samples (20 g) were centrifuged (4500 r/min, 15 min, 4 °C), and the resulting supernatant was weighed. WHC was calculated as follows: WHC (%) = [(sample mass − supernatant mass)/sample mass] × 100%.

### 2.5. Determination of Nutritional Quality

Protein content was determined by the Kjeldahl method. Fat content was measured using the Soxhlet extractor method. The total polysaccharide content in sour camel milk was quantified by the phenol-sulfuric acid method. Free amino acids were analyzed via the ninhydrin reaction. The carbohydrate content was calculated, and the calculation formula is as follows: Carbohydrate = 100% − (Water% + Protein% + Fat% + Ash% + Dietary Fiber%).

### 2.6. Textural Analysis

The textural properties of fermented milk were measured using a Texture Analyzer (TMS-PRO, Beijing Yingsheng Hengtai Technology Co., Ltd., Beijing, China, USK). The TA11/1000 probe was utilized for sample testing, and the test speed was set at 0.50 mm/s, pre-test speed at 2 mm/s, and post-test speed at 0.50 mm/s, respectively. The trigger force was 7 g. The hardness, maximum adhesion, adhesion, and adhesive of the samples were calculated using Texture Expert Excede (vs.1.0) software.

### 2.7. Rheological Analysis

The rheological properties of sour camel milk were measured at 25 °C using a DHR rheometer (TA Instruments, New Castle, DE, USA). A 2 mL sample was evenly spread between two PP-50 parallel plates, followed by amplitude scanning at a constant frequency within the linear viscoelastic region (LVR). The strain was increased from 0.1% to 100% at a constant frequency of 1 Hz, and shear rate scanning was conducted from 0.1 to 700 s^−1^ at 100 rad/s to study the variation of apparent viscosity with shear rate. All measurements were repeated three times for each sample.

### 2.8. Sensory Evaluation

Fourteen candidates (7 males and 7 females) were recruited from local universities. They were aged between 22 and 45. They underwent sensory training and evaluated each sample of sour camel milk in terms of texture, taste, aroma, color, and overall acceptability. The sensory quality was scored based on a 20-point scale (20–16 = excellent, 15–11 = good, 10–6 = satisfactory, 5–1 = moderately satisfactory, 0 = unsatisfactory). All the samples were displayed in transparent containers, and the evaluators would rinse their mouths between each assessment.

### 2.9. Identification of Component Ⅰ Substances

An appropriate amount of sample was weighed into a 2 mL centrifuge tube, as well as 600 µL of methanol containing 2-chloro-L-phenylalanine (4 ppm). It was vortexed for 30 s. Stainless steel grinding beads were added to the tube. This was processed in a tissue homogenizer at 55 Hz for 60 s. The homogenate was subjected to ultrasonic treatment at room temperature for 15 min and centrifuged at 12,000 rpm (4 °C) for 10 min. The supernatant was filtered through a 0.22 μm membrane. The filtrate was added to the test vial for LC-MS detection.

### 2.10. Determination of Volatile Flavor Compounds

Following the methodology described by Martin et al. [22], sour camel milk metabolites were extracted from each group and set aside. Refer to the method of Santos et al. [23] to establish the chromatographic and mass spectrometric conditions. The mass spectrometry transfer line temperature was set at 250 °C, the ion source temperature at 250 °C, the electron impact ionization source at 70 eV, the detector voltage at 1960 V, and the mass scan range from 35 *m*/*z* to 550 *m*/*z*.

### 2.11. Determination of Non-Volatile Flavor Compounds

Referring to the method of Dunn et al. [24], sour camel milk metabolites were extracted from each group and set aside. Reference the parameters and method settings of Zelena et al. [25] to establish the chromatographic and mass spectrometric conditions. For high-performance liquid chromatography, in the positive ion mode, the mobile phase was 0.1% formic acid acetonitrile and 0.1% formic acid water. The negative ion mode with the mobile phase consisted of acetonitrile and 5 mM ammonium formate aqueous solution. Referring to the parameters and methods used by Warwick B et al. [26], the spray voltage for the positive ion mode of the mass spectrometry detector was 3.50 kV, and the voltage of the negative ion spray was −2.50 kV. The primary ion scanning range was 100 *m*/*z*~1000 *m*/*z*, with a secondary fragmentation collision energy of 30 eV and a secondary resolution of 17,500.

### 2.12. Statistical Analysis

SPSS 26.0, Origin 2021, and Metabolic Analyzer were used for statistical analysis and graphing. *p* < 0.05 was considered statistically significant. All experiments were performed in triplicate, and data are presented as mean ± standard deviation (SD). In this study, the visual representations, such as radar charts, heat maps, interaction networks, volcano plots, PCA, and ROAV, were all created through a website (https://www.biodeep.cn/home/tool, 7 February 2025). The instruments used for the determination of non-volatile flavor compounds are the liquid chromatograph (Thermo Fisher Scientific, Bremen, Germany) and the mass spectrometer (Thermo Fisher Scientific, Bremen, Germany), while the instruments used for the determination of volatile flavor compounds are the gas chromatograph (Agilent Technologies, Shanghai, China) and the mass spectrometer (LECO Corporation, St. Joseph, MI, USA).

## 3. Results

### 3.1. Physicochemical Properties of Sour Camel Milk

TPC has a significant impact on the quality of sour camel milk and can confer numerous health benefits to the consumers [27]. Different components of *Sparassis crispa* can increase the total probiotic count in sour camel milk, indicating a synergistic effect between its components and the probiotics in camel milk (Table 1). Component I increased the TPC in fermented camel milk the most, followed by Component Ⅱ. This may be due to the fact that Component I is a *Sparassis crispa* alcohol-soluble polysaccharide, which LAB hydrolyzes to formate, folates, and carbon dioxide. These substances favor the growth of *Streptococcus thermophilus* and *Lactobacillus bulgaricus* [28].

From the results in Table 1, it can be seen that, except for OCS, the acidity of the sour camel milk treated with *Sparassis crispa* and its extract has significantly increased. This might be due to the fact that *Sparassis crispa* and its extracts promoted the growth of acid-producing bacteria. Both FCS and OCS demonstrated significantly improved WHC in sour camel milk stability assays (Table 1). It is likely that the majority of the alcohol-soluble polysaccharides from *Sparassis crispa* are small-molecule polysaccharides, such as dextran. As a result of polysaccharide–protein complex interactions between small-molecule polysaccharide compounds and casein, the internal gel matrix of acid camel milk is strengthened, which reduces whey separation and improves WHC. The OCS is a substantial insoluble dietary fiber that exhibits affinity with casein, forming stable soluble compounds. This interaction also contributes to the enhancement of hydrophobic interactions and hydrogen bonding of protein networks. Furthermore, the crude fiber acts as a structural scaffold, increasing apparent viscosity and stabilizing the casein network through physical entrapment mechanisms.

### 3.2. Nutritional Quality Analysis of Sour Camel Milk

**Figure 2:**
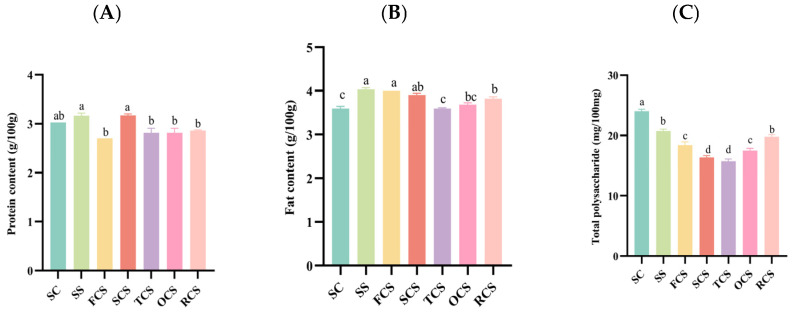

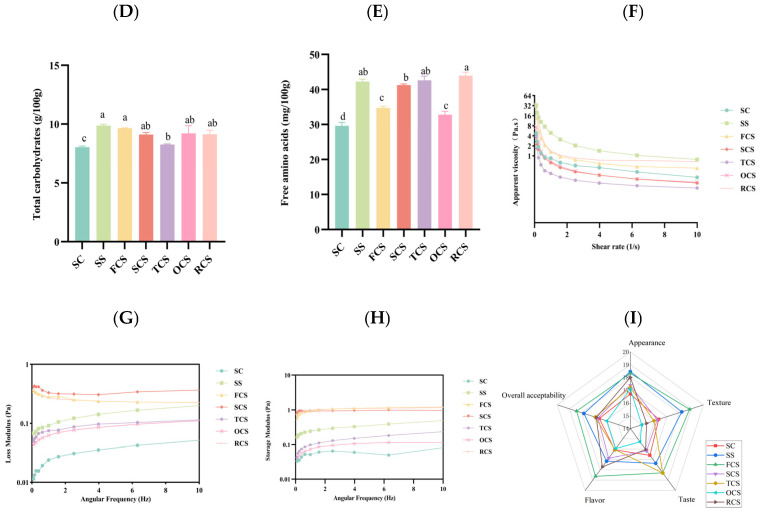
Effect of *Sparassis crispa* components on the quality of sour camel milk. (**A**) Protein content; (**B**) fat content; (**C**) total polysaccharide; (**D**) total carbohydrates; (**E**) free amino acids; (**F**) apparent viscosity; (**G**) loss modulus; (**H**) storage modulus; (**I**) sensory evaluation. Values are expressed as mean ± standard deviation, and for the same parameter, different lowercase letters (a–d) indicate significant differences (*p* < 0.05).

Different fractions of *Sparassis crispa* with sour camel milk resulted in a significant increase in carbohydrate content and free amino acid content and a decrease in total polysaccharide content (Figure 2A–E). It is worth noting that the effects of FCS and SCS on the nutritional quality of sour camel milk fluctuated greatly. The protein content of these two groups showed no significant difference compared to that of SC. However, the fat content increased by 11.11% and 8.33%, the carbohydrate content increased by 20.17% and 13.29%, the free amino acid content increased by 17.26% and 13.29%, and the total polysaccharide content decreased by 13.73% and 32.20%. The result may be that *Sparassis crispa* polysaccharides are preferentially degraded to monosaccharides during fermentation. Some of these monosaccharides are involved in amino acid synthesis via transamination, while the rest are converted to lipids via the acetyl-CoA pathway, resulting in a multistage utilization pattern of the carbon source [29,30,31,32].

### 3.3. Analysis of the Textural Composition of Sour Camel Milk

Texture parameters are key parameters for assessing oral perception and swallowing function [33]. Overall, the addition of different fractions of *Sparassis crispa* increased the hardness, adhesion, maximum adhesion, and adhesive strength of sour camel milk. FCS had the highest indexes and was not significantly different from SS in terms of maximum adhesion, adhesion, and gluing, indicating that the addition of Component I could effectively improve the organizational structure of sour camel milk (Table 2). On the one hand, Component I makes the internal gel structure of sour camel milk more compact and orderly, thereby increasing the internal viscosity [31]. On the other hand, during fermentation of sour camel milk, Component I promotes the growth of more probiotic bacteria, which in turn facilitates the production of extracellular polysaccharides, further increasing the viscosity. Consistent with the results of holding capacity, Component I significantly improved the structural stability of sour camel milk.

### 3.4. Rheological Compositional Analysis of Sour Camel Milk

Apparent viscosity is an important index reflecting the internal structural properties of sour camel milk. The determination of apparent viscosity can determine the curdling condition of sour camel milk and further analyze the texture stability of sour camel milk. Overall, the apparent viscosity decreased as the shear rate increased (Figure 2F–H). As shear continues to increase, noncovalent bonds in the protein network structure are disrupted, and intermolecular electrostatic repulsion and hydrophobic interactions are weakened. The storage modulus (G′) represents the elastic properties of the sample, indicating its ability to recover under external pressure. The loss modulus (G″) represents the viscous properties of the sample, reflecting its resistance to flow. All groups of samples showed G′ greater than G″, indicating that all groups of sour camel milk were in a semi-solid flow state. Specifically, under low shear conditions, the apparent viscosities of sour camel milk with FCS, SS, and RCS were similar, and all were better than those of the other fractions. This suggests that the addition of FCS could enhance the viscosity of sour camel milk, probably because the polysaccharides in it acted as thickeners. The numerical data of the apparent viscosity and the specific values of G′ and G″ at different shear rates for each group are shown in Tables S1–S3. The apparent viscosity of SC was slightly higher than that of SCS, OCS, and TCS, which may be due to the fact that crude fiber is rich in pectin fiber and other dietary fibers. The high methoxyl pectin in it forms a gel under acidic conditions, which alters the structure of acid camel milk, increasing its hardness and viscosity and significantly reducing whey release [34].

### 3.5. Sensory Score Analysis of Sour Camel Milk

The incorporation of exogenous substances significantly influences the sensory properties of foods (Figure 2I). The sensory attributes of sour camel milk, including appearance, texture, taste, and flavor, were all influenced by the addition of different component substances of *Sparassis crispa*. In terms of appearance and texture, SS and RCS exhibited a more appealing pale yellow color. OCS appeared to be partially precipitated, which could be attributed to the fact that most of its components are insoluble dietary fibers, which could not be fully utilized by the probiotics during fermentation. Regarding overall acceptability, taste, and flavor, FCS was the most popular sour camel milk among all groups, with a silky mouthfeel, delicate texture, sweet and sour taste, strong lactic flavor, and moderate mushroom flavor. The same result was also obtained in this study. The addition of the alcoholic extract of *Agaricus bisporus* increased the sensory evaluation score of fermented milk. These findings align with the study by Francisco et al. [35], who reported that the addition of *Agaricus bisporus* alcoholic extract enhanced the sensory scores of fermented milk. Notably, FCS achieved a total sensory evaluation score of 90.24, which exceeded the SS group’s score of 89.12. This indicates that the single-component FCS formulation may possess greater consumer appeal than the full-component SS formulation.

In conclusion, FCS exhibited favorable quality characteristics in terms of total viable bacterial count, texture, and sensory evaluation, and the total score of sensory evaluation was particularly outstanding. Therefore, the subsequent metabolomics study will focus on the FCS.

### 3.6. Component I Substance Identification Analysis

The principal components of Component I were identified as LysoPC(0_0_18_2(9Z,12Z)), LysoPC(18_1(11Z)_0_0), N-(2-hydroxymethyl-3-chloro-4-hydroxyphenyl)anthranilic acid, and 4,4′-methylenebis(2-chloroaniline) in Tables S4 and S5. Among them, the relatively high quantitative value is LysoPC(0_0_18_2(9Z,12Z)). According to the description in the HMDB database, the core of the signal pathway of LysoPC(0_0_18_2(9Z,12Z)) lies in the cascade reaction of G protein–PLC–DAG/IP3–Ca^2+^/PKC–MAPK, which involves various physiological processes such as cell metabolism, immune response, proliferation, and apoptosis. The surface activity of LysoPC(18_1(11Z)_0_0) helps stabilize milk fat globules, prevent lipid stratification, and improve the taste. This chemically diverse profile establishes a foundation for targeted development and utilization strategies.

### 3.7. Analysis of the Flavor Metabolites in Sour Camel Milk

**Figure 3:**
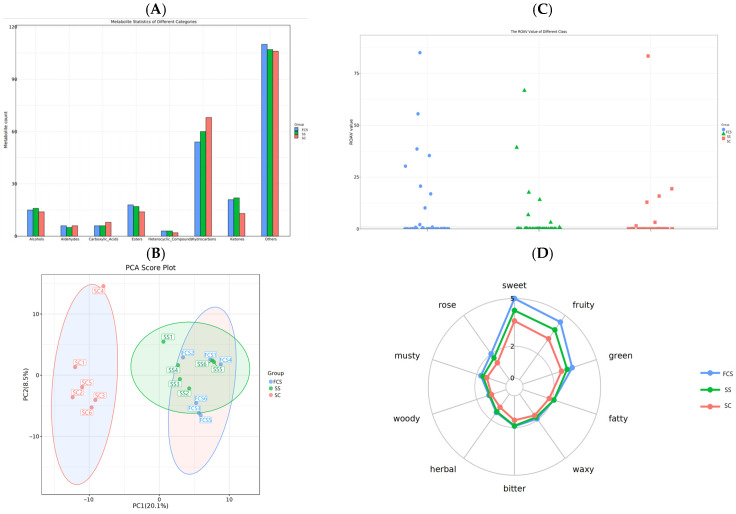

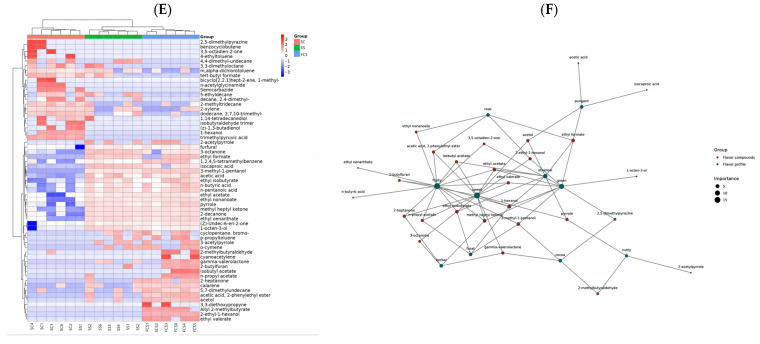
Impact of Component I on flavor metabolites in sour camel milk. (**A**) Bar chart of flavor metabolites category distribution; (**B**) PCA score plot; (**C**) scatter plot of ROAV; (**D**) radar chart of sensory flavor attributes; (**E**) heat map of volatile flavor substances; (**F**) correlation heatmap between sensory attributes and flavor compounds.

The detected flavor compounds were taxonomically annotated using PubChem and Classyfire for chemical classification (Figure 3A). Compared with SC, FCS has added eight ketone, four ester, one alcohol, and one heterocyclic compound, while reducing 14 hydrocarbons. In addition, the rise in esters potentially increases the esterification in order to improve the product flavor [36]. These results indicate that Component I accelerates the interconversion of flavor substances. Compared to the SS, the FCS had an increase of one aldehyde and one ester, but lacked six sugars, one alcohol, and one ketone. The difference in metabolites between the FCS and SS was one, which suggests that it is in the volatiles that the gap between the FCS and SS is smaller [37].

Data were analyzed via PCA to evaluate clustering patterns and dispersion among samples. As shown in Figure 3B, the closer spatial proximity of sample points corresponds to higher similarity in variable compositions [38]. The R^2^ value of the sample model in this study was 0.558, which exceeded 0.5, indicating high reliability of the data. SC were located in the negative semiaxis and FCS and SS in the positive semiaxis, indicating that Component I had a significant effect on sour camel milk. Moreover, the overlapping substances between FCS and SS suggested their main components were similar. To obtain more reliable intergroup differences, PLS-DA analysis was performed. A greater horizontal distance between samples indicates larger intergroup variation, while a closer vertical distance suggests better intra-group reproducibility. As shown in Table S1, significant separation was observed among the three groups, indicating distinct differences between the added components. In this experiment, R^2^Y = 0.993 > 0.5 and Q^2^ = 0.856 > 0.4, demonstrating the reliability of the model.

The flavor intensity of samples from different groups was evaluated by using the Relative Odor Activity Value (ROAV) [39]. As shown in Figure 3C, the ROAV of FCS was higher than that of SC, indicating that the addition of Component I was beneficial in increasing the fermentation flavor intensity of sour camel milk. Interestingly, FCS’s superior flavor intensity aligns with sensory evaluations, where it received higher acceptability scores than SS. Analysis of key flavor compounds revealed distinct profiles: The FCS contained characteristic metabolites, including 2-amylfuran (green vegetable note), isoamyl alcohol (malty sweetness), 2-methylbutyraldehyde (almond-like), and ethyl-1-hexanol (rose-like aroma), whereas SS primarily exhibited 2-amylfuran and diacetyl (creamy) (Table S6). Despite shared dominant compounds between SS and FCS, the FCS demonstrated higher ROAV contributions from multiple odorants, particularly enhancing sweet notes and floral–almond complexity in sour camel milk. These results confirm that co-fermentation with Component I facilitates the formation of diverse aromatic compounds.

Taste and olfactory attributes of different sample groups were analyzed to construct sensory radar charts for flavor profiling. As depicted in Figure 3D, the sensory radar plot shows that FCS exhibited significantly higher intensities in sweetness, fruity, rose-like, and herbal attributes compared to SC. Furthermore, FCS surpassed SS in sweetness, fruity, and rose-like attributes, highlighting the critical role of Component I in modulating the flavor profile of sour camel milk.

Key differential metabolites between FCS and other groups were analyzed by hierarchical clustering of relative quantitative values, visualized in a heatmap (Figure 3E). The FCS showed significantly higher abundance of esters (e.g., isobutyl acetate), alcohols (1-Hexanol), and ketones (e.g., 2-heptanone). Esters, synthesized through acid–alcohol esterification, contributed fruity or floral aromas typical of fermented dairy products [36]. Ketones, likely derived from fatty acid oxidation, enhanced creamy or nutty notes in sour camel milk. Conversely, alkanes (3,3-dimethyloctane, 2,4-dimethyl decane) and amides (N-acetylglycinamide) showed negative correlations with FCS but positive correlations with SC. N-Acetylglycinamide, associated with fishy/bitter off-flavors through protein degradation, and 1-hexanol (grassy/alcoholic at high concentrations but fruity at low levels) [40], collectively explained the reduced gaminess and milder flavor profile in FCS. Probiotic-driven metabolic shifts in FCS suppressed these undesirable compounds while promoting ester/ketone biosynthesis, thereby improving overall sensory quality.

To investigate flavor compound–sensory attribute correlations, an interaction network was constructed for the top 10 sensory attributes (Figure 3F). Component I enhanced fruity aroma, primarily attributed to metabolites such as ethyl heptanoate, ethyl nonanoate, acetic acid, 2-phenylethyl ester, 2-butylfuran, and 3-octanone. Concurrently, it elevated sweetness through metabolites such as 2-ethyl-1-hexanol, ethyl acetate, ethyl valerate, and 2-heptanone. Component I enhances the sour camel milk flavor through two pathways: partial retention of its flavor compounds (e.g., phenylacetaldehyde [floral/honey], styrene [sweet], phenol [sour]) during fermentation enriches the profile, as evidenced by eight shared metabolites in FCS. Simultaneously, microbial and chemical conversion of precursors generates active flavor compounds, optimizing sensory complexity [41]. This dual mechanism elevated the sensory quality of FCS and SS over SC, supported by glycoside hydrolysis, lipid oxidation, and Strecker degradation during co-fermentation.

### 3.8. Analysis of the Non-Volatile Flavor Metabolites in Sour Camel Milk

**Figure 4:**
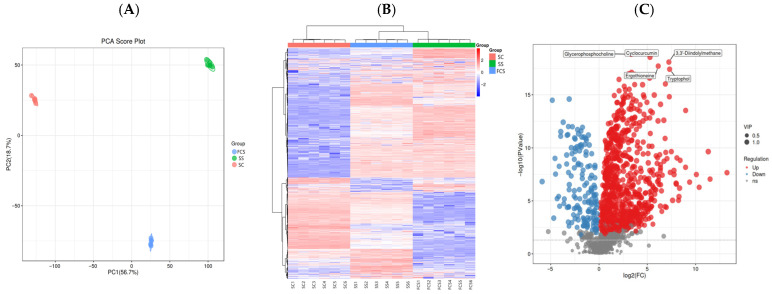

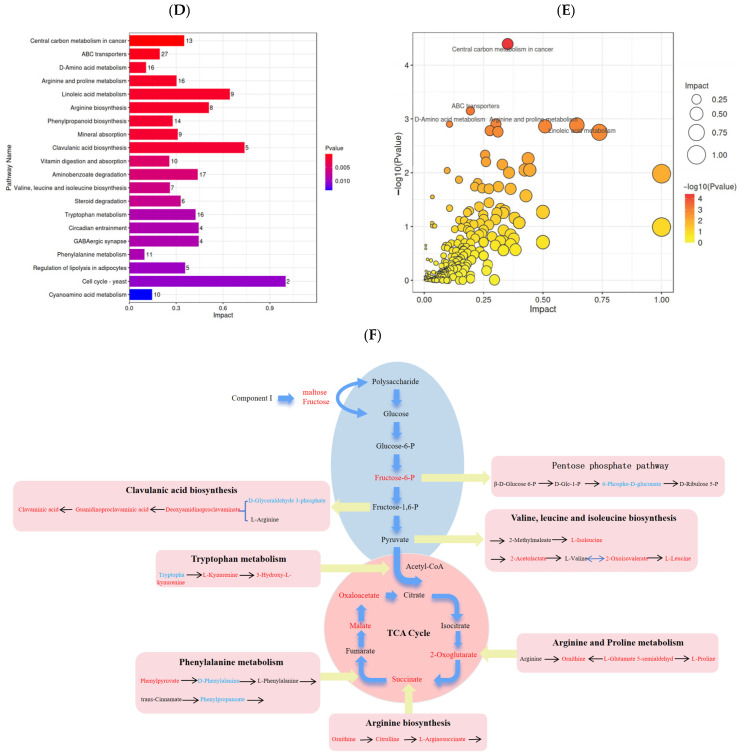
Identification of non-volatile metabolites in sour camel milk. (**A**) PCA score plot; (**B**) heatmap of differential metabolites; (**C**) volcano plot of differential metabolites; (**D**) bar plot of pathway; (**E**) scatter plot of pathway analysis; (**F**) impact of variation map on core metabolic pathways.

To comprehensively assess non-volatile metabolite profiles across FCS, SC, and SS, principal component analysis (PCA) based on non-volatile metabolites showed distinct clustering patterns (Figure 4A). SC clustered on the negative axis, while FCS and SS occupied the positive axis, indicating substantial compositional divergence induced by Component I addition.

Hierarchical clustering heatmap (Figure 4B) of normalized metabolite profiles demonstrated closer similarity between FCS and SS at the primary classification level, suggesting Component I likely accounts for the majority of SS’s functional effects. Volcano plot analysis revealed Component I differential regulation of non-volatile metabolites (Figure 4C). Compared to SC, FCS exhibited 740 upregulated and 148 downregulated metabolites, indicating enhanced metabolic complexity. FCS vs. SS showed milder shifts (308 upregulated/282 downregulated).

Component I significantly altered key metabolite profiles in fermented camel milk, with FCS’s top 20 metabolites upregulated 165- to 9731-fold (Table S7), notably, 9α-hydroxyandrosta-1,4-diene-3,17-dione (9731.81-fold) and cholesterol (165.03-fold), both critical for steroid catabolism. Argininosuccinic acid (2545-fold) was cleaved by argininosuccinate lyase (ASL) into arginine and fumarate—the latter suppressing hypertension via KCNMB1 downregulation—while reducing glutathione (GSH) and elevating malondialdehyde (MDA) in brain tissues. TCA cycle intermediates–succinic acid (311-fold) and oxalacetic acid (217-fold)–enhanced flavor complexity and surface tension, critical for food additive applications, and mediated central carbon metabolism in cancer pathways [42]. Pantothenic acid (323-fold) amplified vitamin/CoA biosynthesis, improving nutritional profiles. Component I promoted probiotic-driven macromolecule breakdown into small metabolites, accelerating nutrient cycling and metabolic health through lipid-lowering and glucose-modulating effects.

Metabolite downregulation in Component I modulated sour camel milk revealed the top 20 downregulated metabolites with reductions ranging from 9- to 50-fold, including pseudoephedrine (33-fold decrease), a key intermediate in probiotic alkaloid biosynthesis (Table S8). Component I was shown to block pseudoephedrine conversion to methamphetamine, mitigating health risks [43]. Isoeugenol (25-fold decrease), a potent spice allergen restricted in industrial applications, aligned with safety regulations [44]. Significant reductions were observed in cGMP, a secondary messenger regulating ion channels through interactions with cGMP-dependent protein kinases and phosphodiesterases (PDEs) [45]. Sulfate suppressed membrane transport and monoamide synthesis, indicating disrupted nutrient cycling. Notably, coumesterol (12.5-fold reduction)—a broad-spectrum antimicrobial against *E. coli* and *B. subtilis*—demonstrated reduced activity, likely due to LAB-driven depletion of sulfur compounds under Component I-enriched conditions [46]. These shifts suggest that Component I reshapes microbial ecology: enhanced LAB proliferation competitively consumes substrates critical for spoilage bacteria, while suppressing pathways linked to harmful metabolite synthesis. The resultant metabolic reconfiguration promotes probiotic dominance and optimizes fermented camel milk’s safety and functional properties.

The top 20 metabolic pathways of probiotics post-Component I supplementation (Figure 4D) spanned four KEGG categories: metabolism, human diseases, cellular processes, and environmental transport. Key pathways included membrane transport (27 metabolites), amino acid metabolism (56 metabolites), lipid metabolism (9 metabolites), digestive system (9 metabolites), and cellular processes (16 metabolites). Notably, 87 metabolites were linked to amino acid metabolism, indicating Component I enhances proteolytic degradation to support bacterial growth [47]. Newstead [48] emphasized amino acid prioritization for improved nutrient assimilation and digestive efficiency.

Metabolic pathway analysis revealed significant enrichment differences between FCS and SS, where proximity to the upper-right quadrant in Figure 4E indicates stronger pathway impact. Component I exhibited marked upregulation (red-labeled) in central carbon metabolism in cancer, ABC transporters, D-amino acid metabolism, arginine and proline metabolism, and linoleic acid metabolism, highlighting its role in energy metabolism. These two amino acid-related pathways further indicate enhanced proteolytic activity.

Differential metabolites were mapped to the KEGG database, with a focus on amino acid metabolism (Figure 4F and Table S9). Organic oxidants in Component I, such as aminofructose-6-phosphate and 1-[(5-amino-5-carboxypentyl)amino]-1-deoxyfructose, increased the accumulation of polysaccharides (e.g., sucrose, lactose) in sour camel milk, promoting hydrolysis to elevate fructose 6-phosphate levels and accelerate the glycolytic cycle. The pentose phosphate pathway triggered substantial depletion of 6-phospho-D-gluconate, while 1,6-fructose bisphosphate was linked to clavulanic acid biosynthesis via N2-(2-carboxyethyl)-arginine synthase. Subsequent amino acid biosynthesis pathways were mediated by pyruvate (valine/leucine/isoleucine), acetyl-CoA (tryptophan), α-ketoglutarate (arginine/proline), and succinate (arginine/phenylalanine) enhanced levels of phenylalanine, leucine, isoleucine, L-proline, L-argininosuccinate, and tryptophan [49]. In Component I, betaine accelerated L-proline conversion to L-glutamate, while oxoadipic acid supplied energy for acyl-CoA synthesis via tryptophan degradation [44,48]. Residual amino acids from unutilized Component I further contributed to this enrichment, including L-isoleucine, L-proline, L-arginine, and L-tryptophan. Notably, leucine, tryptophan, and tyrosine were identified as key enhancers of probiotic growth, which may explain the elevated LAB viability during co-fermentation [50]. Arginine, critical for hydrogen bonding and protein acid interactions, participates in diverse metabolic reactions across biological systems [51,52]. Phenylalanine, an aromatic amino acid, is hydroxylated to tyrosine by phenylalanine hydroxylase, supporting neurotransmitter/hormone synthesis and regulating glucose homeostasis [53].

## 4. Conclusions

The results of this study indicate that Component I has a significant effect on improving the quality of camel milk. Specifically, Component I increased the total viable count of probiotics in camel milk fermentation by 2.67 times, and improved the stability of camel milk from 28.97% to 38.03%. In sensory evaluations conducted by professionals, Component I had a better sensory evaluation for the co-fermentation of camel milk (92.50 points) than the full-component SS group (89.01 points). The GC-MS results showed that Component I significantly increased the contents of 2-undeanone, 2-heptanone, 1-octen-3-ol, and ethyl caprylate in acid camel milk, enhancing the aroma of acid camel milk, and reducing n-acetylglycinamide, 3,3-dimethyloctane, decane, 2,4-dimethyl, and other alkanes and amine substances, thereby improving the off-flavor of acid camel milk. The LC-MS results indicated that most of the upregulated metabolites were related to amino acids, free fatty acids, organic acids, and sugars, and up to 87 metabolites were related to the differential metabolic pathways of amino acids. Component I could regulate the glycolysis, pyruvate cycle, valeric acid synthesis, TCA cycle, valine, leucine, and isoleucine biosynthesis, tryptophan metabolism, arginine and proline metabolism, arginine biosynthesis, phenylalanine metabolism, and other pathways of probiotics in acid camel milk to improve the quality of acid camel milk. In conclusion, Component I of *Sparassis crispa* improved the quality and flavor of acid camel milk. However, this study was conducted in a laboratory setting, and further research is needed in industrial production.

## Figures and Tables

**Table 1:** Total number of lactic acid bacteria (LAB), titratable acidity (TA), and water-holding capacity (WHC) of sour camel milk with different components of *Sparassis crispa*.

Groups	Total Number Of Viable Bacteria (LgCFU/mL)	Acidity (°T)	Water-Holding Capacity (%)
SC	9.13 ± 0.02 ^f^	88.31 ± 1.22 ^e^	28.97 ± 0.52 ^e^
SS	9.83 ± 0.02 ^a^	104.60 ± 0.70 ^b^	48.99 ± 1.14 ^a^
FCS	9.56 ± 0.03 ^c^	109.70 ± 0.87 ^a^	38.03 ± 1.20 ^c^
SCS	9.46 ± 0.01 ^d^	90.01 ± 0.81 ^de^	31.35 ± 0.79 ^de^
TCS	9.29 ± 0.01 ^e^	91.81 ± 0.16 ^d^	33.22 ± 0.07 ^d^
OCS	9.25 ± 0.02 ^e^	86.49 ± 0.17 ^f^	39.12 ± 0.47 ^bc^
RCS	9.73 ± 0.03 ^b^	98.10 ± 1.42 ^c^	42.23 ± 0.08 ^b^

Note: Values are expressed as mean ± standard deviation. For the same parameter, different lowercase letters (a–f) in the same column indicate significant differences (*p* < 0.05). SC, sour camel milk; SS, *Sparassis crispa* sour camel milk (2% *w*/*v*); FCS, Component I of sour camel milk (0.59% *w*/*v*); SCS, Component II of sour camel milk (0.15% *w*/*v*); TCS, Component III of sour camel milk (0.05% *w*/*v*); OCS, Component IV of sour camel milk (1.21% *w*/*v*); RCS, Component V of sour camel milk (2% *w*/*v*).

**Table 2:** Textural composition of sour camel milk with components extracted from *Sparassis crispa*.

Groups	Hardness/N	Maximum Adhesion/N	Adhesion/mJ	Adhesive/N
SC	0.322 ± 0.021 ^c^	0.062 ± 0.015 ^b^	0.217 ± 0.025 ^d^	0.105 ± 0.014 ^c^
SS	0.473 ± 0.014 ^a^	0.133 ± 0.011 ^a^	0.536 ± 0.017 ^a^	0.197 ± 0.008 ^a^
FCS	0.413 ± 0.016 ^b^	0.118 ± 0.011 ^a^	0.487 ± 0.010 ^ab^	0.178 ± 0.010 ^ab^
SCS	0.370 ± 0.025 ^bc^	0.110 ± 0.012 ^a^	0.480 ± 0.021 ^ab^	0.158 ± 0.011 ^b^
TCS	0.412 ± 0.010 ^b^	0.102 ± 0.006 ^a^	0.357 ± 0.019 ^c^	0.145 ± 0.012 ^b^
OCS	0.368 ± 0.021 ^bc^	0.062 ± 0.018 ^b^	0.297 ± 0.036 ^c^	0.111 ± 0.008 ^c^
RCS	0.458 ± 0.012 ^a^	0.113 ± 0.006 ^a^	0.434 ± 0.028 ^b^	0.179 ± 0.011 ^ab^

Note: Values are expressed as mean ± standard deviation. For the same parameter, different lowercase letters (a–d) in the same column indicate significant differences (*p* < 0.05). SC, sour camel milk; SS, *Sparassis crispa* sour camel milk (2% *w*/*v*); FCS, Component I of sour camel milk (0.59% *w*/*v*); SCS, Component II of sour camel milk (0.15% *w*/*v*); TCS, Component III of sour camel milk (0.05% *w*/*v*); OCS, Component IV of sour camel milk (1.21% *w*/*v*); RCS, Component V of sour camel milk (2% *w*/*v*).

## Data Availability

The original contributions presented in this study are included in the article/Supplementary Materials. Further inquiries can be directed to the corresponding authors. The data is kept in the School of Research Center of JUNCAO Technology, Fujian Agriculture and Forestry University.

## Supplementary Materials

The following supporting information can be downloaded at https://www.mdpi.com/article/10.3390/foods14173042/s1. Table S1. The apparent viscosity of each group at different shear rates; Table S2. The numerical data of the specific values of G′ for each group; Table S3. The numerical data of the specific values of G″ for each group; Table S4: Quantitative identification table of main substance categories of Component I; Table S5: Quantitative identification table of main components of Component I; Table S6. The top 9 FCS, SS, and SC flavor metabolites; Table S7: The top 20 metabolites upregulated in FCS and SC; Table S8: The top 20 metabolites downregulated in FCS and SC. Table S9. The top 20 metabolic pathways between FCS and SC; Figure S1. PLS-DA.

## Abbreviations

Lactic acid bacteria (LAB); water-holding capacity (WHC); sour camel milk (SC); *Sparassis crispa* sour camel milk (SS); Component Ⅰ of sour camel milk (FCS); Component Ⅱ of sour camel milk (SCS); Component Ⅲ of sour camel milk (TCS); Component Ⅳ of sour camel milk (OCS); Component Ⅴ of sour camel milk (RCS); Total Plate Count (TPC); titratable acidity (TA); linear viscoelastic region (LVR).
