# Supplementary material for: Improvement of Quality of Sour Camel Milk by Extract of Sparassis crispa: Physicochemical Properties, Sensory Quality and Metabolic Changes
Source: Foods. 2025 Aug 29;14(17):3042. doi: 10.3390/foods14173042 (PMC12428353; doi:10.3390/foods14173042)

Supplementary Materials

Table S1. The apparent viscosity of each group at different shear rates

Shear rate (s^{-1})	SC	SS	FCS	SCS	TCS	OCS	RCS
0.100008	4.73557	32.4422	31.444	4.45566	3.89433	7.31718	37.9551
0.158482	2.72177	19.48	13.4631	2.19615	1.67698	3.89311	20.1012
0.251188	1.84148	14.1693	5.70295	1.55054	0.876693	2.49304	10.2105
0.398102	1.2145	10.3792	3.42057	1.14148	0.536958	1.34333	4.69768
0.630929	0.901324	7.51249	2.0982	0.836465	0.361278	0.965359	2.23499
0.99995	0.854227	4.90526	1.32793	0.622942	0.297377	0.67108	1.38956
1.58483	0.627865	3.08381	0.948472	0.449058	0.230232	0.484719	1.05392
2.5118	0.516388	2.04368	0.756728	0.335121	0.185637	0.350407	0.858145
3.98095	0.447011	1.42167	0.593073	0.265553	0.153842	0.263034	0.733062
6.30941	0.331251	1.03262	0.481309	0.201095	0.128277	0.202859	0.724999
9.99972	0.227879	0.776962	0.431244	0.15384	0.108849	0.161298	0.668933
15.8485	0.173086	0.603076	0.436216	0.125855	0.0940984	0.132128	0.486952
25.1181	0.137498	0.479295	0.353323	0.105962	0.0831506	0.110801	0.345457
39.8093	0.112502	0.387027	0.239053	0.0918372	0.0751323	0.0951112	0.248888
63.0934	0.0956757	0.316513	0.179579	0.0808281	0.0672502	0.0836367	0.191097
99.9965	0.0827151	0.263024	0.143033	0.0716919	0.0607191	0.075612	0.151097
158.484	0.0734253	0.226129	0.117488	0.0655684	0.0584371	0.0706133	0.124601

Table S2. The numerical data of the specific values of G' for each group

Angular Frequency(Hz)	SC	SS	FCS	SCS	TCS	OCS	RCS
100	-7.38253	12.2449	1.60638	-7.78219	7.10248	-1.9269	2.01825
63.0957	-2.90109	5.28933	1.47361	-2.39131	2.95257	-0.710771	1.57705
39.8105	-1.08064	2.39796	1.38436	-0.193724	1.29243	-0.166376	1.37123
25.1188	-0.369537	1.20448	1.32218	0.649026	0.627391	0.0322916	1.26955
15.849	-0.104039	0.712423	1.26602	0.871538	0.357439	0.0998192	1.20774
10.0001	0.0813138	0.491703	1.21592	0.967491	0.236545	0.113111	1.16653
6.30957	0.0492714	0.388106	1.16525	0.981553	0.184165	0.115089	1.13238
3.98105	0.0591433	0.327278	1.11637	0.94932	0.150744	0.106149	1.09912
2.51189	0.0644703	0.294703	1.07147	0.93887	0.130248	0.0946661	1.06328
1.5849	0.0603149	0.26613	0.993998	0.934176	0.111351	0.0854357	1.02863
1	0.0509572	0.242842	0.956692	0.901812	0.0981932	0.0732582	0.990445
0.630957	0.051588	0.226606	0.90076	0.866025	0.0856143	0.0629865	0.943729
0.398107	0.0436827	0.209544	0.834089	0.913244	0.0738066	0.0551782	0.88258
0.251189	0.0357153	0.195822	0.755902	0.923423	0.0638055	0.048267	0.707736
0.15849	0.034868	0.181574	0.687067	0.846097	0.055203	0.0418919	0.60044
0.1	0.033426	0.163186	0.567854	0.80564	0.0430952	0.0377012	0.751135

Table S3. The numerical data of the specific values of G'' for each group

Angular Frequency(Hz)	SC	SS	FCS	SCS	TCS	OCS	RCS
100	0.489004	0.524841	0.227508	0.855418	0.300177	0.471383	0.239377
63.0957	0.18236	0.447631	0.22313	0.586211	0.269411	0.281622	0.232078
39.8105	0.104707	0.373139	0.219213	0.483707	0.202629	0.185625	0.226158
25.1188	0.0760445	0.301321	0.218816	0.444726	0.154186	0.0110547	0.223448
15.849	0.063409	0.246407	0.220259	0.399947	0.133915	0.098404	0.223659
10.0001	0.0516076	0.198756	0.223276	0.366228	0.114393	0.110908	0.224646
6.30957	0.0422958	0.166803	0.229819	0.34267	0.103426	0.0966949	0.229599
3.98105	0.0353275	0.141978	0.236305	0.305569	0.0971014	0.0856447	0.237671
2.51189	0.0306734	0.122049	0.250616	0.312637	0.0872393	0.0770112	0.248142
1.5849	0.027149	0.105649	0.285721	0.316276	0.077016	0.0699754	0.257564
1	0.0239067	0.0915452	0.27941	0.330932	0.0748295	0.0624023	0.275546
0.630957	0.0191757	0.0847247	0.295117	0.3624	0.0703702	0.0567084	0.294023
0.398107	0.0154817	0.0813196	0.314816	0.417727	0.0670181	0.0523084	0.32088
0.251189	0.0156353	0.0730701	0.332741	0.415811	0.0589163	0.0476605	0.321239
0.15849	0.0132641	0.067733	0.353302	0.428707	0.0549141	0.0461997	0.336388
0.1	0.01147412	0.0681329	0.354327	0.418192	0.0496234	0.0429372	0.363811

Table S4. Quantitative Identification Table of Main Substance Categories of Component I

Class	Relative quantitative values（$\times 10^9$）
Glycerophospholipids	91.47
Carboxylic acids and derivatives	40.71
Benzene and substituted derivatives	29.19
Fatty Acyls	26.74
Prenol lipids	24.69
Organooxygen compounds	21.55
Organonitrogen compounds	13.57
Keto acids and derivatives	10.42
Indoles and derivatives	8.77
5'-deoxyribonucleosides	8.40

Table S5. Quantitative Identification Table of Main Components of Component I

	Name	Relative quantitative values （$\times 10^{9}$）	Molecular weight	Molecular formula	Class
1	LysoPC(0_0_18_2(9Z, 12Z))	62.25	519.33	$C_{26}H_{50}NO_7P$	Glycerophospholipids
2	LysoPC(18_1(11Z)_0_0)	13.33	521.35	$C_{26}H_{52}NO_7P$	Glycerophospholipids
3	N-(2-hydroxymethyl-3-chloro-4-hydroxyphenyl)anthranilic acid	10.79	293.05	$C_{14}H_{12}ClNO_4$	Benzene and substituted derivatives
4	4,4'-Methylenebis(2-chloroaniline)	10.76	266.04	$C_{13}H_{12}Cl_2N_2$	Benzene and substituted derivatives
5	2-hydroxyphytanic acid	8.66	328.29	$C_{20}H_{40}O_3$	Prenol lipids
6	Oxoadipic acid	8.23	160.04	$C_6H_8O_5$	Keto acids and derivatives
7	Betaine	8.11	117.08	$C_5H_{11}NO_2$	Carboxylic acids and derivatives
8	5'-Methylthioadenosine	8.01	297.09	$C_{11}H_{15}N_5O_3S$	5'-deoxyribonucleosides
9	Butyrylcarnitine	7.45	231.15	$C_{11}H_{21}NO_4$	Fatty Acyls
10	Phytosphingosine	7.26	317.29	$C_{18}H_{39}NO_3$	Organonitrogen compounds
11	Malic acid	6.78	134.02	$C_4H_6O_5$	Hydroxy acids and derivatives
12	LysoPC(16_0_0_0)	6.62	495.33	$C_{24}H_{50}NO_7P$	Glycerophospholipids
14	(+)-threo-2-Amino-3,4-dihydroxybutanoic acid	5.67	135.05	$C_4H_9NO_4$	Carboxylic acids and derivatives
15	Mevalonic acid-5P	4.53	228.04	$C_6H_{13}O_7P$	Organic phosphoric acids and derivatives
17	6-Hydroxy-8-docosanone	3.97	340.33	$C_{22}H_{44}O_2$	Fatty Acyls
18	Tryptophol	3.94	161.08	$C_{10}H_{11}NO$	Indoles and derivatives
19	Dimethylpropiothetin	3.51	134.04	$C_5H_{10}O_2S$	Carboxylic acids and derivatives
20	1-Phenylethylamine	3.22	121.09	$C_8H_{11}N$	Organonitrogen compounds

Table S6. The top 9 FCS, SS, and SC flavor metabolites

	Name	Formula	SC (Relative value)	SS (Relative value)	FCS (Relative value)	OdorCharacter
1	isoamyl alcohol	$C_5H_{12}O$	12.91	6.80	30.27	sweet, malty, rancid,rubber,
2	2-ethyl-1-hexanol	$C_8H_{18}O$	0.00	0.00	20.64	Rose, Green
3	2-methylbutyraldehyde	$C_5H_{10}O$	0.23	0.18	38.56	Cocoa, Almond
4	acetic acid	$C_2H_4O_2$	3.21	17.65	35.38	pungent,vinegar
5	2-undecanone	$C_{11}H_{22}O$	0.14	3.18	10.14	Orange, Fresh, Green
6	methyl heptyl ketone	$C_9H_{18}O$	0.00	0.01	0.01	Fruity, Floral, Fatty
7	diacetyl	$C_4H_6O_2$	83.39	66.74	16.91	pleasant, buttery
8	isovaleric acid	$C_5H_{10}O_2$	15.85	14.14	55.50	Rancid Cheese, Sweaty, Putrid
9	2-amylfuran	$C_9H_{14}O$	19.39	39.29	85.01	Green Beans, Vegetable

Table S7. The top 20 metabolites up-regulated in FCS and SC

	Name	Mz	FC	Pos /neg	FCS_Mean	SC_Mean
1	9-α-Hydroxyandrosta-1,4-diene-3,17-dione	323.16	9731.81	pos	1297654621.16	133341.48
2	Avocadene 2-acetate	351.25	2888.61	pos	482424467.72	167009.49
3	Argininosuccinic acid	271.10	2545.47	neg	4347576421.52	1707963.99
4	Dapdiamide A	284.15	1322.35	pos	5705901510.66	4314964.82
5	Trans-zeatin riboside	308.17	1082.54	pos	540773880.71	499539.94
6	Bisphenol B	260.13	666.72	pos	40059937.42	60085.03
7	3-Dimethylallyl-4-hydroxyphenylpyruvate	266.14	501.61	pos	592561199.15	1181315.89
8	N-Succinyl-L,L-2,6-diaminopimelate	274.09	496.72	pos	370886191.87	746666.83
9	4,7-Dihydroxy-2H-1-benzopyran-2-one	161.08	338.92	pos	594872718.02	1755186.42
10	Pantothenic acid	218.10	323.24	neg	74565468.17	230683.16
11	Succinic acid	179.02	311.08	neg	62448946.24	200747.24
12	D-1-[(3-Carboxypropyl)amino]-1-deoxyfructose	266.12	300.01	pos	324131354.56	1080389.95
13	Pinosylvin methyl ether	244.13	266.15	pos	45979208.28	172757.83
14	P-Coumaraldehyde	295.10	256.02	neg	77873287.05	304173.85
15	DL-Mannitol	181.07	252.82	neg	3175131615.25	12558907.40
16	Vestitol	333.09	218.92	neg	107412689.87	490651.50
17	Oxalacetic acid	263.01	217.43	neg	93668714.01	430795.55
18	Beta-Glucogallin	377.05	196.47	neg	19519240.19	99349.71
19	5-Methoxytryptamine	173.11	179.55	pos	101482666.87	565215.63
20	Cholesterol	369.35	165.03	pos	96348247.84	583818.65

Table S8. The top 20 metabolites down-regulated in FCS and SC

	Name	Mz	FC	Pos /neg	FCS_Mean	SC_Mean
1	Trihomomethionine	236.09	0.02	neg	2237783.89	132297491.03
2	Pseudoephedrine	122.13	0.03	pos	5960095.67	170381811.96
3	Isoeuchinol	121.10	0.04	pos	7121073.37	167979376.62
4	Roquefortine D	374.20	0.04	pos	14596646.10	331090691.86
5	Cyclic GMP	344.04	0.04	neg	1239604.86	32091022.72
6	Acetyl-N-formyl-5-methoxykynurenamine	247.11	0.05	pos	4354717.46	87985361.16
7	L-Alanyl-gamma-D-glutamyl-L-lysine	347.19	0.06	pos	131585169.42	2064064112.26
8	Protomycinolide IV	371.22	0.06	pos	159686211.11	2698423663.97
9	Terpendole E	460.29	0.06	pos	114936330.69	2086509139.89
10	Nonactin	759.42	0.06	pos	6657645.75	116286481.80
11	Sulfate	194.93	0.06	neg	7627124.34	118348838.56
12	2-Aminoacrylic acid	110.02	0.08	pos	360511840.11	4488294837.41
13	4,21-dehydrogeissoschizine	352.17	0.08	pos	6285574.81	75685533.52
14	Coumesterol	249.02	0.08	neg	1692420.40	22221639.58
15	Syoyualdehyde	225.11	0.10	pos	1396027.46	14216712.75
16	Gingerol	295.19	0.10	pos	46487608.04	462526277.44
17	Inosinic acid	332.06	0.10	pos	5347732.12	52661750.56
18	L-Threonine	164.06	0.10	neg	2506320.21	25610256.08
19	3-ureido-isobutyrate	130.07	0.11	pos	9475846.71	83371672.75
20	Milnacipran	230.16	0.11	pos	5323840.76	49597145.63

Table S9. The top 20 metabolic pathways between FCS and SC

	pathway_name	Pvalue	Compound_name
1	Central carbon metabolism in cancer	0.000	L-Glutamic acid; Oxoglutaric acid; Oxalacetic acid; Succinic acid; L-Serine; L-Methionine; L-Tryptophan; Fructose 6-phosphate; L-Leucine; L-Histidine; L-Proline; Malic acid; L-Isoleucine
2	ABC transporters	0.001	Phosphate; L-Glutamic acid; Sulfate; L-Serine; L-Ornithine; Urea; Glycerol 3-phosphate; L-Leucine; L-Histidine; L-Proline; L-Threonine; Adenosine; Riboflavin; Inosine; Uridine; Guanosine; DL-Mannitol; L-Isoleucine; Cytidine; Trimethylglycine; Sorbitol; D-methionine; Deoxycytidine; Phthalic acid; 4-O-alpha-D-Galactopyranuronosyl-D-galacturonic acid; Deoxyinosine; FAMP
3	D-Amino acid metabolism	0.001	L-Glutamic acid; Oxoglutaric acid; Oxalacetic acid; L-Serine; L-Methionine; L-Ornithine; L-Histidine; L-Proline; Phenylpyruvic acid; L-Threonine; 5-Aminopentanoic acid; N-Acetyl-L-glutamic acid; meso-Diaminoheptanedioate; D-methionine; D-phenylalanine; 1-Pyrroline-4-hydroxy-2-carboxylate
4	Arginine and proline metabolism	0.001	AdoMet; L-Glutamic acid; L-Ornithine; Urea; L-Proline; 5-Aminopentanoic acid; N-Carbamoylputrescine; L-Aspartate-semialdehyde; Spermine; Creatinine; 4-Guanidinobutanoic acid; L-Glutamic gamma-semialdehyde; 4-Acetamidobutanoic acid; Tiformin; Gamma-glutamyl-L-putrescine; gamma-Glutamyl-gamma-aminobutyraldehyde
5	Linoleic acid metabolism	0.001	Arachidonic acid; Linoleic acid; FA 18_2; 13-L-Hydroperoxylinoleic acid; 13-HODE; alpha-Dimorphecolic acid; 9(S)-HPODE; 9,12,13-TriHOME; 9,10,13-TriHOME
6	Arginine biosynthesis	0.001	L-Glutamic acid; Oxoglutaric acid; L-Ornithine; Urea; Citrulline; N2-Acetylornithine; N-Acetyl-L-glutamic acid; Argininosuccinic acid
7	Phenylpropanoid biosynthesis	0.002	Coniferyl alcohol; 4-Hydroxycinnamic acid; Sinapine; Sinapyl alcohol; Coniferaldehyde; p-Coumaraldehyde; 4-Hydroxystyrene; Methyleugenol; Isoeugenol; 5-Hydroxyconiferaldehyde; tricoumaroyl spermidine; triferuloyl spermidine; trihydroxyferuloyl spermidine; coniferyl acetate
8	Mineral absorption	0.002	Phosphate; L-Serine; L-Methionine; L-Tryptophan; L-Leucine; Galactose; L-Proline; L-Threonine; L-Isoleucine
9	Clavulanic acid	0.002	Glyceraldehyde 3-phosphate; Deoxyamidinoproclavaminate;

	biosynthesis		Amidinoproclavaminate; Proclavaminate; Dihydroclavaminate
10	Vitamin digestion and absorption	0.005	Pyridoxal 5'-phosphate; Flavin mononucleotide; Niacinamide; Cholesterol; Pyridoxal; Riboflavin; Pyridoxamine 5'-phosphate; Pantothenic acid; Menadione; Vitamin D3
11	Aminobenzoate degradation	0.005	Phenol; 4-Hydroxybenzoic acid; Benzaldehyde; Quinone; Benzoyl-CoA; 4-Hydroxybenzaldehyde; Vanillin; 4-Nitrophenol; Mandelic acid; aminophenol; 1,3,5-Trihydroxybenzene; 4-Nitrocatechol; 4-Aminophenol; Hydroxylaminobenzene; FT-0776012; Diethylthiophosphate; Aminohydroquinone
12	Valine, leucine and isoleucine biosynthesis	0.006	L-Leucine; alpha-Ketoisovaleric acid; L-Threonine; L-Isoleucine; Citraconic acid; Isopropylmaleic acid; (S)-2-Acetolactate
13	Steroid degradation	0.007	Cholesterol; 3,4-Dihydroxy-9,10-secoandrosta-1,3,5(10)-triene-9,17-dione; ST 19_4;O6; 9alpha-Hydroxyandrosta-1,4-diene-3,17-dione; Secophenol; Boldione
14	Tryptophan metabolism	0.009	L-Tryptophan; L-Kynurenine; Indoleacetaldehyde; Serotonin; Tryptophol; N-Acetylserotonin; Melatonin; aminophenol; Indole-3-acetamide; 3-Indoleacetonitrile; L-3-Hydroxykynurenine; indole-3-glycol aldehyde; Acetyl-N-formyl-5-methoxykynurenamine; 6-HYDROXYMELATONIN; 5-Methoxytryptamine; S-(Indolylmethylthiohydroximoyl)-L-cysteine
15	GABAergic synapse	0.009	L-Glutamic acid; Oxoglutaric acid; Succinic acid; Cyclic AMP
16	Circadian entrainment	0.009	L-Glutamic acid; Cyclic AMP; Cyclic GMP; Melatonin
17	Phenylalanine metabolism	0.009	Succinic acid; Phenylpyruvic acid; Benzoyl-CoA; p-Hydroxyphenylacetic acid; 3-Oxoadipyl-CoA; D-phenylalanine; 2-Phenylacetamide; N-Acetyl-L-phenylalanine; Phenylethylamine; 3-phenyllactic acid; Hydrocinnamic acid
18	Regulation of lipolysis in adipocytes	0.010	Adenosine; Arachidonic acid; Cyclic AMP; Cyclic GMP; Corticosterone
19	Cell cycle - yeast	0.010	Phosphate; Cyclic AMP
20	Cyanoamino acid metabolism	0.013	L-Serine; L-Isoleucine; Linamarin; Acetone cyanohydrin; Dhurrin; gamma-Glutamyl-beta-cyanoalanine; Lotaustralin; Benzeneacetonitrile; SCHEMBL157788; (1Z,2S)-2-methylbutanal oxime

Figure S1. PLS-DA

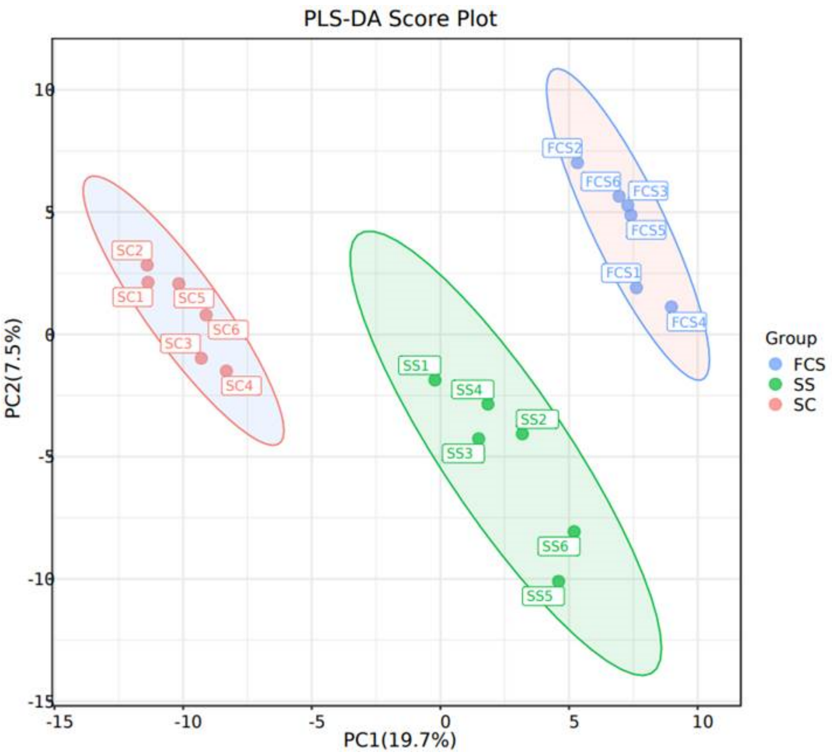

Supplement: Supplementary file 1 [file foods-14-03042-s001.zip › foods-3820997-supplementary.pdf]
